# Caffeine-Induced Global Reductions in Resting-State BOLD Connectivity Reflect Widespread Decreases in MEG Connectivity

**DOI:** 10.3389/fnhum.2013.00063

**Published:** 2013-03-04

**Authors:** Omer Tal, Mithun Diwakar, Chi-Wah Wong, Valur Olafsson, Roland Lee, Ming-Xiong Huang, Thomas T. Liu

**Affiliations:** ^1^Center for Functional MRI, University of California San DiegoLa Jolla, CA, USA; ^2^Department of Bioengineering, University of California San DiegoLa Jolla, CA, USA; ^3^Department of Radiology, University of California San DiegoLa Jolla, CA, USA; ^4^Research and Radiology Services, VA San Diego Healthcare SystemSan Diego, CA, USA

**Keywords:** magnetoencephalography, fMRI, BOLD, resting-state, functional connectivity, caffeine

## Abstract

In resting-state functional magnetic resonance imaging (fMRI), the temporal correlation between spontaneous fluctuations of the blood oxygenation level dependent (BOLD) signal from different brain regions is used to assess functional connectivity. However, because the BOLD signal is an indirect measure of neuronal activity, its complex hemodynamic nature can complicate the interpretation of differences in connectivity that are observed across conditions or subjects. For example, prior studies have shown that caffeine leads to widespread reductions in BOLD connectivity but were not able to determine if neural or vascular factors were primarily responsible for the observed decrease. In this study, we used source-localized magnetoencephalography (MEG) in conjunction with fMRI to further examine the origins of the caffeine-induced changes in BOLD connectivity. We observed widespread and significant (*p* < 0.01) reductions in both MEG and fMRI connectivity measures, suggesting that decreases in the connectivity of resting-state neuro-electric power fluctuations were primarily responsible for the observed BOLD connectivity changes. The MEG connectivity decreases were most pronounced in the beta band. By demonstrating the similarity in MEG and fMRI based connectivity changes, these results provide evidence for the neural basis of resting-state fMRI networks and further support the potential of MEG as a tool to characterize resting-state connectivity.

## Introduction

The temporal correlation between spontaneous low-frequency fluctuations in the blood oxygenation level dependent (BOLD) signal measured using functional magnetic resonance imaging (fMRI) is being increasingly used to characterize functional connectivity (FC) in the brain. Functional connectivity MRI (fcMRI) was first demonstrated by Biswal et al. (1995), who observed synchronous BOLD fluctuations within the motor cortex during rest. Subsequent studies found additional resting-state networks such as the visual network (Lowe et al., 1998), the default mode network (Raichle et al., 2001), the task positive network (Fox et al., 2005), and a number of task-related networks (Smith et al., 2009). fcMRI studies are advancing our understanding of the brain’s behavioral states (Fox et al., 2007; He et al., 2007) and pathology (Lowe et al., 2002; Greicius et al., 2004; Lui et al., 2008; Kwak et al., 2010), and have also proven to be useful for the assessment of cognitive performance (Hampson et al., 2006; Song et al., 2008a).

In most fcMRI studies, changes in FC measures are interpreted as evidence of underlying changes in neuronal connectivity. However, as the BOLD signal reflects both vascular and neural factors, the interpretation of resting-state FC observations can be challenging. The BOLD response is a complex function (known as the hemodynamic response) of changes in oxygen metabolism (CMRO_2_), cerebral blood flow (CBF) and blood volume, and thus provides an indirect measure of the underlying neuro-electrical activity (Buxton et al., 2004). Other non-neuronal confounds, such as metabolic and vascular factors rising from differences in age, diet, medications, and pathology, can alter the neurovascular coupling linking neural activity to the observed hemodynamic changes (Cohen et al., 2002; D’Esposito et al., 2003; Liu et al., 2004; Behzadi and Liu, 2005; Liau et al., 2008) and thus affect the BOLD signal. Hence, changes in metabolic and vascular factors can give rise to changes in FC measures even when there is no underlying change in neural connectivity.

Magnetoencephalography (MEG) is a non-invasive brain imaging modality which can aid in the interpretation of fcMRI measures (Hamalainen et al., 1993). MEG avoids the hemodynamic confounds of the BOLD signal by providing a direct measure of neuro-electromagnetic activity. Furthermore, the temporal resolution of the MEG signal is on the timescale of neural firing events, permitting a substantially wider frequency range for activity analysis than the fMRI signal, whose temporal resolution is limited by the temporal broadening inherent in the hemodynamic response (Buxton et al., 2004). Compared to electroencephalography (EEG), MEG provides superior spatial resolution, due in large part to its robustness to the conductivity profile of the human head. In addition, the high number of sensors and availability of advanced reconstruction algorithms (Robinson and Vrba, 1998; Wipf et al., 2010) have enabled more accurate characterization of the underlying neuro-electromagnetic sources. In recent years, MEG has emerged as a valuable tool in the investigation of connectivity and power fluctuations in both studies of healthy volunteers (de Pasquale et al., 2010; Liu et al., 2010; Brookes et al., 2011a; Mantini et al., 2011) and patients suffering from neurophysiological disorders such as autism, Alzheimer’s disease, Parkinson’s disease, and stroke (Gomez et al., 2011; Schoonheim et al., 2011; Zamrini et al., 2011; Tarapore et al., 2012a,b; Westlake et al., 2012). Perhaps most notably, Brookes et al. (2011b) recently used MEG and beamforming algorithms to validate the electrophysiological basis of the resting-state fcMRI networks.

In order to compare changes in fMRI and MEG FC, it is useful to have a pharmacological agent that can alter the state of connectivity in healthy subjects for a period of time (e.g., an hour or more) that is sufficiently long to facilitate experimental measurements. Caffeine is a widely used stimulant that reliably perturbs the neural and vascular systems of the brain for several hours or more (Fredholm et al., 1999). We have previously shown that a 200-mg dose of caffeine significantly reduced resting-state BOLD connectivity in the motor cortex (Rack-Gomer et al., 2009) as well as in a global fashion across the brain (Wong et al., 2012). Caffeine constricts the vascular system and decreases CBF by antagonizing adenosine A_2_ receptors (Fredholm et al., 1999) and stimulates the neural system through antagonism of adenosine A_1_ receptors (Dunwiddie and Masino, 2001). Both pathways can alter the measured BOLD signal, where the vascular pathway does so by modifying the mechanisms of neurovascular coupling and thus the overall hemodynamic response function, while the neural pathway can modulate the input to the response function. It has been demonstrated that caffeine significantly reduces baseline CBF (Liau et al., 2008; Rack-Gomer et al., 2009) and increases baseline CMRO_2_ (Griffeth et al., 2011), a combination which tends to increase the BOLD response to an arbitrary neural input. On the other hand, it has also been shown that caffeine tightens the coupling between CBF and CMRO_2_, reducing the BOLD sensitivity to neural activity (Chen and Parrish, 2009; Griffeth et al., 2011). These two effects tend to cancel out, resulting in little or no impact on the task-related BOLD response (Laurienti et al., 2002; Liau et al., 2008; Chen and Parrish, 2009; Griffeth et al., 2011). As task-related and resting-state BOLD responses are likely to share the same underlying hemodynamic pathways, it is unlikely that vascular and metabolic changes are the primary mechanisms behind the observed reductions in BOLD connectivity. With regards to caffeine’s effect on neural activity, several studies find that caffeine reduces EEG power (Dimpfel et al., 1993; Siepmann and Kirch, 2002) and may also decrease inter-hemispheric coherence (Reeves et al., 2002). Based on these prior findings, we hypothesized that the caffeine-induced reductions in BOLD connectivity are mainly driven by decreases in neural connectivity, and that MEG measures of FC would show a similar caffeine-related decrease. To test our hypothesis, we conducted a double-blind placebo-controlled study with a repeated measures design in which MEG and fMRI resting-state data were collected on subjects both prior to and after the ingestion of caffeine (or placebo). At present, there is not a clear consensus among resting-state FC studies with respect to the state of the eyes during the experiment, with some studies employing an eyes closed (EC) protocol while others use an eyes open (EO) with fixation protocol (Liu et al., 2010; Van Dijk et al., 2010). In light of this situation, we chose to compare the effects of caffeine on fMRI and MEG connectivity in both states.

## Materials and Methods

### Experimental protocol

Twelve healthy volunteers were initially enrolled in this study after providing informed consent. Two subjects were not able to complete the study due to excessive motion and dental artifacts, resulting in a final sample size of 10 subjects (four males and six females; ages 21–33 years; mean of 25.6 years). To minimize potential confounds due to differing levels of caffeine consumption (Jones et al., 2000; Reeves et al., 2002), we recruited subjects with low levels of caffeine usage (<50 mg/day). Participants were instructed to abstain from caffeine for 24 h prior to being scanned, as well as to maintain low caffeine consumption for a 2-month period prior to the beginning of the study and throughout the entire duration of the study.

The study employed a double-blind, placebo-controlled, repeated measures design. For each modality (MEG and fMRI), each subject participated in two independent imaging sessions, a control session and a caffeine session, where the order of the two sessions was random. Each of the four imaging sessions (MEG control and caffeine; fMRI control and caffeine) was separated from the other sessions by at least 2 weeks. Half the subjects started with MEG sessions while the other half started with fMRI sessions. Each session consisted of a pre-dose section and a post-dose section, with each MEG and fMRI section lasting about 30 and 60 min, respectively. After the pre-dose section, subjects were taken out of the MEG or MRI scanner and asked to ingest a capsule containing 200 mg of caffeine or placebo. A 40-min period was allotted between capsule ingestion and the first functional scan of the post-dose section, as previous studies have shown that the absorption of caffeine from the gastrointestinal tract reaches 99% about 45 min post ingestion (Fredholm et al., 1999).

Each MEG scan section consisted of four 5 min resting-state scans, two with EC and two with EO, in the following order: EC, EO, EC, and EO. Subjects were instructed to stay awake, relax, and think of nothing in particular (Stamatakis et al., 2010; van den Heuvel and Hulshoff Pol, 2010) while keeping their hands open, laying flat. During EO resting-state scans, participants were asked to visually fixate on a black cross placed on a white screen, while during the EC resting-state scans they were asked to keep their eyes closed and to imagine the black cross. Each fMRI scan section included a high-resolution anatomical scan, two 5 min resting-state scans, one EO scan and one EC scan, and additional scans described below and in Wong et al. (2012). The instructions given to the subjects for the resting-state scans were the same as those used for the MEG sessions. The order of the EC and EO fMRI resting-state scans was randomized.

### Data acquisition

#### Magnetoencephalography

Magnetoencephalography data were measured using an Elekta/Neuromag™ whole-head MEG system with 204 gradiometers and 102 magnetometers in a magnetically shielded room (IMEDCO-AG, Switzerland). Electro-oculogram (EOG) electrodes were used to record eye blinks and movements. Data were sampled at 1000 Hz and pre-processed using MaxFilter (Neuromag™) to detect and correct for saturated and spurious channels, suppress magnetic interference from inside and outside the sensor array, and compensate for disturbances due to magnetic material in the region of the head (Taulu et al., 2004; Taulu and Simola, 2006; Song et al., 2008b, 2009). As MaxFilter is limited in certain artifact-removal tasks (e.g., eye movement), we also applied temporal independent components analysis (ICA) to the data using the fast ICA algorithm (Hyvarinen, 1999) to remove notable residual artifacts due to eye movements, eye blinks, and cardiac activity. The independent components to be removed were selected by visual inspection of their temporal and spatial signatures (e.g., the EOG time-course was used for visual comparison), typically removing one to three components in a given dataset.

#### Functional magnetic resonance imaging

A detailed description of the acquisition and analysis of the fMRI data was previously provided in Wong et al. (2012). For convenience, we restate the relevant details in this and subsequent sections. Imaging data were acquired using a 3-T GE Discovery MR750 whole body system with an eight-channel receiver coil. High-resolution anatomical data were collected using a magnetization prepared 3D fast spoiled gradient (FSPGR) sequence (TI = 600 ms, TE = 3.1 ms, FOV = 25.6 cm, 256 × 256 × 176 matrix, slice thickness = 1 mm, and flip angle = 8°). Whole brain BOLD resting-state data were acquired using an echo planar imaging (EPI) sequence (TR = 1.8 s, TE = 30 ms, FOV = 24 cm, 64 × 64 matrix, slice thickness = 4 mm, slice gap = 1 mm, # of slices = 30, and flip angle = 70°). Field maps were acquired using a gradient recalled acquisition in steady state (GRASS) sequence (TE_1_ = 6.5 ms, TE_2_ = 8.5 ms), with equivalent in-plane parameters and slice coverage as in the BOLD data, and the phase difference between the two echoes was used to correct the BOLD data for magnetic field inhomogeneities (Jenkinson, 2003; Fessler et al., 2005). Cardiac pulse and respiratory data were monitored using a pulse oximeter (InVivo Corp.) which was placed on the subject’s finger and a respiratory effort transducer (BIOPAC) placed around the abdomen. The physiological data were sampled at 40 Hz using a multi-channel data acquisition board (National Instruments).

### Data processing

#### Magnetoencephalography

Using the high-resolution anatomical data obtained in the MRI scan, a boundary element based triangular mesh of 5-mm mesh size was generated for each subject from their inner-skull surface. FreeSurfer was used to define a fixed source grid (7 mm spacing) on the brain’s gray–white matter boundary, which was then divided into cortical regions of interest (ROI) using the FreeSurfer computed parcellations (Desikan et al., 2006). With the inner-skull triangular mesh and gray matter source grid, the MEG forward model calculation for the lead-field (gain) matrix was performed using a boundary element model (Mosher et al., 1999; Huang et al., 2007). Registration of MRI and MEG data was performed using positioning information obtained with a Polhemus Isotrak system prior to each MEG session.

In our analysis, we considered MEG data both within a wide-band range of 1–50 Hz and within the following bands: delta (δ) – 1–4 Hz, theta (θ) – 4–8 Hz, alpha (α) – 8–13 Hz, low and high beta (β) – 13–20 and 20–30 Hz, respectively, and low gamma (γ) – 30–50 Hz. The frequency filtered MEG data were then projected into source space using the array-gain constraint minimum-variance regularized vector beamformer (van Drongelen et al., 1996; Van Veen et al., 1997; Robinson and Vrba, 1998; Sekihara and Nagarajan, 2008), yielding a set of band-limited time-courses for each source location. Covariance matrices were generated independently for each frequency band and experimental run using all 300 s of the recorded data from each run. The regularization level was set uniquely for each individual MEG recording by utilizing a modified “broken-stick” model as described in Behzadi et al. (2007), which helps to identify the meaningful (data-related) principal components. A statistical distribution of expected eigenvalues, derived from random normally distributed data with rank and Frobenius norm equal to that of the MEG data of interest, was used for comparison and determination of the noise level [i.e., the number of significant (*p* < 0.05) modes]. The value of the first non-significant (noise) component then represented the cut-off and was used as the regularization parameter. For each frequency band of interest, the source time-courses were Hilbert transformed to construct the corresponding analytic signals. The envelope of oscillatory power fluctuations (also referred to as the “Hilbert envelope”) was obtained via computation of the amplitude of the analytic signal (Brookes et al., 2004, 2012a). Temporal smoothing was applied following the approach of Brookes et al. (2011a) where an “average Hilbert envelope” time-course was obtained by dividing the envelope time-course into 500 ms blocks and averaging the envelope within each block. These average Hilbert envelope time-courses were then used for the connectivity computations described below, yielding the Correlation of Average Envelopes as defined in Brookes et al. (2011a).

#### Functional magnetic resonance imaging

Anatomical data were skull-stripped and segmented into white matter, gray matter, and cerebral spinal fluid using FSL (Smith et al., 2004). The post-dose anatomical volume was registered to the pre-dose volume using AFNI (Cox, 1996), and the resulting rotation and shift parameters were applied to the post-dose functional data. A binary brain mask was created using the skull-stripped anatomical data. For each slice, the mask was eroded by two voxels along the border to eliminate voxels at the edge of the brain (Rack-Gomer and Liu, 2012). The first six time points of fMRI data were discarded to allow magnetization to reach steady state. Nuisance terms were removed from fMRI data by means of multiple linear regression using the following regressors: linear and quadratic trends, six motion parameters, RETROICOR (Glover et al., 2000) and RVHRCOR (Chang and Glover, 2009) regressors, and the mean BOLD signal calculated from WM and CSF voxels (partial volume threshold of 0.99 for each tissue type). BOLD data were then low pass filtered with a cut-off frequency of 0.08 Hz (Biswal et al., 1997; Cordes et al., 2001; Fox et al., 2005).

### Connectivity measures

For each subject, we used the FreeSurfer cortical parcellations (Desikan et al., 2006) to define anatomical ROIs. As described in Wong et al. (2012), we discarded ROIs for which any subject had less than five voxels within a region, resulting in a total of 40 ROIs (20 per hemisphere). For fMRI data, an average BOLD time-course was calculated for each ROI using all voxels within the region. To reduce spatial leakage effects on the ROI-to-ROI MEG connectivity estimates that are inherent to the beamforming process (Brookes et al., 2012b), we defined a smaller MEG source region within each larger anatomical ROI. A central source for each of the cortical ROIs was defined as the source with the smallest mean path length to all the other sources within the ROI. Next, a sphere-shaped region was defined to include every source that was both within 12 mm of the central source and contained within the same ROI. The average Hilbert envelopes within this region were then averaged to provide a mean MEG time-course for each ROI.

To assess connectivity, we computed the Pearson correlation coefficient (*r*) between the average time-courses for each pair of ROIs (780 pairs). For each modality, the correlation coefficient was computed for each of the four acquisition sections (pre-dose and post-dose sections of both the Control and Caffeine sessions). For the MEG data, the correlation coefficients from repeated scans (e.g., the two pre-dose EC scans) were averaged. For quantitative assessments, the Pearson correlation scores were converted to the Fisher *z*-scores using the Fisher transformation (Luckhoo et al., 2012). The change in the *z*-score metric (Δ*z* = post-dose *z*-score minus pre-dose *z*-score) in each session (caffeine and control) was calculated, and a repeated measures two-way analysis of variance (ANOVA) (Keppel and Wickens, 2004) was then used to examine the effects of two factors on the measured connectivity: (1) the effect of caffeine/control and (2) the effect of ROI pair (Wong et al., 2012).

## Results

### Whole brain connectivity

**Figure 1:**
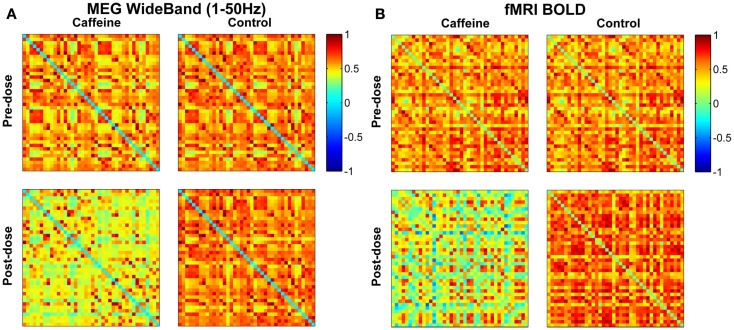
**Connectivity matrices for a representative subject in the eyes closed condition showing Pearson correlations between all pairs of ROIs for the (A) MEG wide-band (1–50 Hz) data and the (B) fMRI BOLD data**. Each entry corresponds to the correlation between one pair of ROIs, with the axes corresponding to the ROI indices (1–40). Both MEG and fMRI connectivity are visibly lower in the post-dose caffeine section than in the pre-dose caffeine section, while no change is apparent in the control session for either modality. ROI labels (1–20 left hemisphere; 21–40 right hemisphere): anterior cingulate, middle frontal, cuneus, fusiform, inferior parietal, isthmus cingulate, lateral orbitofrontal, medial orbitofrontal, pars opercularis, post central, posterior cingulate, precentral, precuneus, rostral anterior cingulate, rostral middle frontal, superior frontal, superior parietal, superior temporal, supramarginal, insula.

For a representative subject, Pearson correlation coefficient matrices indicating the degree of connectivity in the EC condition for all ROI pairs are displayed in Figure 1 for each of the four scan sections (pre-dose and post-dose sections of the caffeine and control sessions). The MEG and fMRI connectivity matrices are shown in the left and the right hand sides of the figure, respectively, and the fMRI matrices are similar to those previously presented in Wong et al. (2012). The MEG connectivity metrics were obtained using the wide-band frequency range (1–50 Hz). MEG correlations in the post-dose caffeine data are visibly lower than in the pre-dose caffeine data, indicating a caffeine-induced global decrease in this subject’s connectivity, while there is not a widespread difference between the pre-dose and post-dose MEG correlations in the control session. A similar qualitative assessment can be made about the fMRI data, where the connectivity in the post-dose section of the caffeine session shows a widespread decrease as compared to the pre-dose condition.

**Figure 2:**
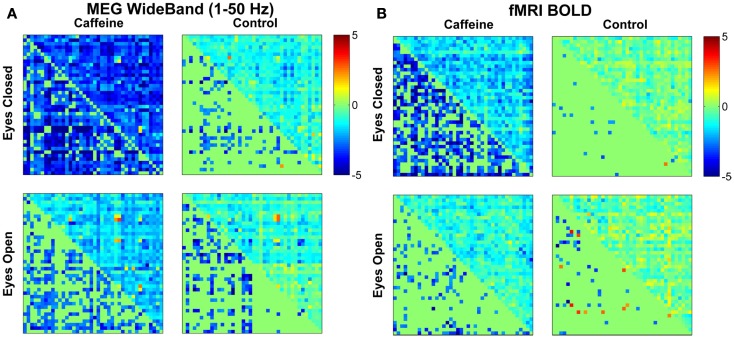
**Changes in mean connectivity between all ROI pairs averaged across the subject group for both the (A) MEG wide-band (1–50 Hz) data and the (B) fMRI BOLD data**. The results from the eyes closed condition are shown in the top row, while the bottom row corresponds to the results from the eyes open condition. Each subplot is divided into an upper triangle which shows the mean change in *z*-scores for each ROI pair and a lower triangle displaying the corresponding *t*-statistic for each ROI pair (in a mirrored fashion where only significant (*p* < 0.05) entries are filled in). A negative value (blue color) corresponds to a caffeine-induced decrease in connectivity, while a positive value (red) corresponds to an increase in connectivity. Qualitative assessment shows broad decreases in connectivity due to caffeine for the eyes closed condition and to a lesser extent in the eyes open condition, for both modalities.

Figure 2 shows the changes in *z*-score (post-dose minus pre-dose) averaged across subjects for both conditions (EC and EO) and sessions (control and caffeine), with the changes for MEG and fMRI shown in the left and the right hand sides of the figure, respectively. The upper triangle of each matrix shows the mean changes in the *z*-score metric (across subjects) for all ROI pairs, while the lower triangle shows the *t*-statistics of those ROI pairs that exhibited a significant (*p* < 0.05) change in connectivity across the sample. Decreases and increases in *z*-scores and *t*-statistics are indicated by blue and red hues, respectively. From a qualitative perspective, broad decreases in MEG and fMRI connectivity can be observed for the EC caffeine data and to a lesser extent in the EO data. The control data for both conditions (EC and EO) shows fewer significant changes than the caffeine data, with the MEG data showing only significant decreases and the fMRI data showing a nearly even mix of increases and decreases.

As a quantitative assessment of the data, Table 1 summarizes the results provided by the two-way repeated measures ANOVA. For the EC condition, the caffeine/control factor showed a significant effect for both MEG and fMRI (*p* ≤ 0.01), indicating that the change in correlation was significantly different between the caffeine and control sessions in both modalities. *Post hoc* two-tailed *t*-tests showed a significant decrease in mean *z*-score averaged across ROI pairs for the caffeine session [*t*(9) = −4.43, *p* = 1.7e−3 for MEG; *t*(9) = −5.63, *p* = 3e−4 for fMRI] whereas significant changes were not observed for the control session [*t*(9) = −1.96, *p* = 0.08 for MEG; *t*(9) = −0.69, *p* = 0.51 for fMRI]. The interaction terms between the factors were not significant for either modality, suggesting that the effect of the caffeine/control factor was largely independent of ROI pair. For both modalities, the effect of the caffeine/control factor did not reach significance in the EO condition. As a result, we will focus on the EC condition for the remainder of the analysis.

**Table 1:** **Quantitative assessment of the group data for both modalities (fMRI and wide-band MEG) and both conditions (eyes closed and open) using repeated measures two-way analysis of variance (ANOVA) to examine the effects of (1) caffeine/control and (2) ROI pair on the measured connectivity changes**.

Factor	Dof	Eyes closed				Eyes open			
		MEG		fMRI		MEG		fMRI	
		*F*	*p*	*F*	*p*	*F*	*p*	*F*	*p*
Caffeine/control	(1, 9)	11.89	<0.01	10.45	0.01	1.38	0.27	2.70	0.13
ROI pairs	(779, 7011)	1.21	<1e−4	1.41	<1e−6	1.87	<1e−6	1.44	<1e−6
Interaction	(779, 7011)	0.85	0.99	1.04	0.23	1.07	0.10	0.99	0.60

*The caffeine/control factor showed a significant (*p* ≤ 0.01) effect in the eyes closed condition but not in eyes open condition (for both modalities). Interaction terms were non-significant, suggesting caffeine/control factor was largely independent of ROI pair*.

### MEG band-specific activity

**Figure 3:**
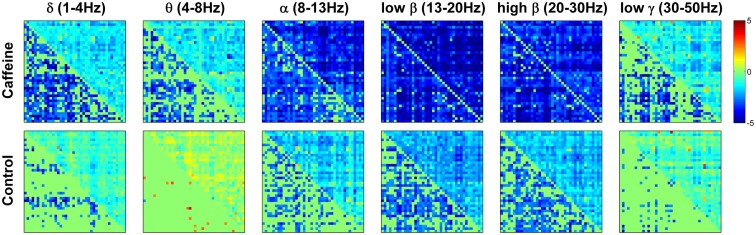
**Changes in mean connectivity (eyes closed condition) between all ROI pairs averaged across the subject group for each of the six MEG frequency bands of interest: δ (1–4 Hz), θ (4–8 Hz), α (8–13 Hz), low β (13–20 Hz), high β (20–30 Hz), and low γ (30–50 Hz)**. Each subplot is created in the same manner as was described in the caption of Figure 2. Widespread decreases in connectivity (*z*-scores) are evident in the caffeine data across all bands, with the strongest reductions appearing in the α and β bands.

To provide further insight into the global MEG connectivity reductions observed in the EC condition, Figure 3 shows the mean connectivity changes across all ROIs for each of the frequency bands defined in the Section “Materials and Methods” (displayed in the same manner as the wide-band MEG data in Figure 2). Widespread decreases in *z*-scores are evident in the caffeine data across all bands, with the strongest reductions appearing in the α, low β, and high β bands. The data from these three bands also showed connectivity decreases in the control session. A quantitative assessment using the two-way repeated measures ANOVA (Table 2) indicates that only the θ, low β, and high β bands showed a significant main effect (*p* ≤ 0.0165) of the caffeine/control factor. The interaction term was not significant (*p* ≥ 0.58) for these bands. Although the α band exhibited qualitatively large reductions in connectivity as well, the effect of the caffeine/control factor was not significant (*p* = 0.085).

**Table 2:** **Quantitative assessment of the group data for each of the six MEG frequency bands of interest (eyes closed condition) using repeated measures two-way analysis of variance (ANOVA) to examine the effects of (1) caffeine/control and (2) ROI pair on the measured connectivity changes**.

Factor	δ		θ		α		Low β		High β		Low γ	
	*F*	*p*	*F*	*p*	*F*	*p*	*F*	*p*	*F*	*p*	*F*	*p*
Caffeine/control	1.72	0.22	14.38	<0.01	3.8	0.085	9.67	0.012	8.64	0.016	1.32	0.28
ROI pairs	0.80	1.00	1.23	<1e−4	2.08	<1e−6	1.66	<1e−6	1.26	<1e−6	1.45	<1e−6
Interaction	0.90	0.98	0.80	1.00	0.92	0.93	0.99	0.58	0.94	0.85	0.87	0.99

*The caffeine/control factor showed a significant (*p* ≤ 0.0165) effect in both the θ and β bands (interaction terms were non-significant; *p* ≥ 0.58)*.

To gain a better understanding of the contribution of different bands to the wide-band MEG connectivity changes, we computed the Pearson correlation between the mean Δ*z*-scores (averaged across all ROIs for each subject) for the wide-band and band-limited MEG data. As shown in Table 3, significant correlations were observed for the α, low β, and high β bands in the caffeine session and for the α, low β, and low γ bands in the control session.

**Table 3:** **Correlation (Pearson) of the connectivity changes in the wide-band MEG to the connectivity changes in each of the six MEG frequency bands of interest (eyes closed condition)**.

		δ	θ	α	Low β	High β	Low γ
Pearson correlation of Δ*z* across all subjects	Caffeine	0.22	0.47	0.84*	0.79*	0.72*	0.40
	Control	0.42	0.57	0.96*	0.76*	0.56	0.67*

*Connectivity changes were quantified for each subject by averaging the change in the z-score metric across all ROI pairs. Significant (*p* < 0.05) correlations are noted with a star*.

### Comparing fMRI and MEG global effects

**Figure 4:**
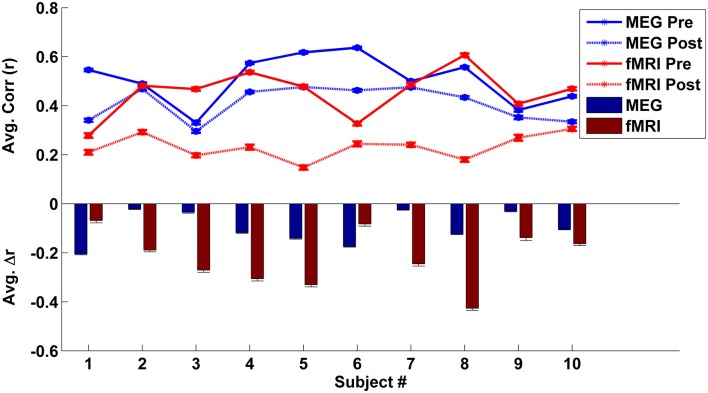
**Top panel –** individual mean global correlations (averaged across all ROI pairs) are plotted for the MEG (blue lines) and fMRI (red line) eyes closed caffeine scans. The solid lines represent the mean global correlation from the pre-dose section while the dotted lines correspond to the post-dose section connectivity. **Bottom panel –** individual caffeine-induced changes in mean global correlation (post-dose section minus pre-dose section) for the MEG (blue) and the fMRI (red) caffeine sessions. Although all subjects exhibited a decrease in overall connectivity for both modalities, the magnitudes of the MEG and fMRI decreases were not significantly related. Error bars represent the standard error across regions.

To estimate each subject’s mean global correlation, we averaged the correlation values across all ROI pairs from their respective connectivity matrix. The mean global correlations for the fMRI and MEG caffeine sessions (pre-dose and post-dose section) for all 10 subjects are plotted in the top panel of Figure 4. The bar graph in the bottom panel of Figure 4 summarizes the caffeine-induced changes (post-dose minus pre-dose) in the MEG and fMRI mean global correlations for each subject. While all subjects exhibited a decrease in their overall connectivity regardless of modality, there was not a significant relation between the magnitude of the MEG and fMRI changes [Pearson correlation coefficient (*r)* = −0.18, *p* = 0.62; Spearman’s rank correlation ρ = −0.12, *p* = 0.73]. Further examination of Figure 4 reveals that 8 out of the 10 subjects showed a larger decrease in correlation in the fMRI data as compared to the MEG data, while the remaining two subjects (numbers 1 and 6) showed a smaller correlation decrease in the fMRI data. These two subjects also exhibited the lowest overall fMRI pre-dose global connectivity (solid red curve in top panel) and the smallest changes in the fMRI mean global correlation when compared to the rest of the group (red bars in bottom panel). Recomputing the correlation between the magnitudes of connectivity changes for the remaining eight subjects results in a larger (although not significant) correlation (Pearson’s *r* = 0.62, *p* = 0.09; Spearman’s ρ = 0.71, *p* = 0.06). The relation between MEG and fMRI measures was similar for the bands which were found in the previous section to have the strongest similarity to the wide-band MEG changes. For example, in the low-beta band, we found Pearson’s *r* = 0.72, *p* = 0.04 and Spearman’s ρ = 0.69, *p* = 0.07.

## Discussion

Caffeine has been previously shown to reduce the connectivity of spontaneous BOLD fluctuations across the brain (Rack-Gomer et al., 2009; Wong et al., 2012). Given the BOLD signal’s complex dependence on both neural and vascular factors, it is not straightforward to assess how caffeine’s modulation of these factors contributes to the observed changes in BOLD connectivity when only fMRI measures are available. In this study, we utilized MEG measures to better determine the contribution of neural changes in connectivity to the observed reductions in BOLD connectivity. We found that caffeine led to a significant and widespread reduction in both fMRI and MEG measures of resting-state connectivity in the EC condition. Neither modality revealed a significant change in connectivity for the EO condition. While our prior study (Wong et al., 2012) suggests that the lack of pronounced connectivity changes in the EO state may reflect a smaller additive global signal component (as compared to the EC state), further studies are needed to determine why both fMRI and MEG connectivity changes are more evident in the EC versus EO state. Overall, our results indicate that caffeine-related changes in neural connectivity (as assessed with MEG) play a substantial role in decreasing BOLD connectivity. In addition, as the widespread decreases in BOLD connectivity have been shown to be related to a decrease in the resting-state fMRI global signal, the concomitant decreases in MEG connectivity provide further evidence for a neural basis to the global signal (Scholvinck et al., 2010; Wong et al., 2012). In this study, we focused on the global nature of the caffeine-induced changes in connectivity. Future studies comparing changes in specific functional networks as well as differences in complex network measures of connectivity (Rubinov and Sporns, 2010) may provide deeper insights into the nature of the caffeine-induced effects and the relation between fMRI and MEG connectivity measures.

While all subjects showed a decrease in both global fMRI and MEG connectivity measures, we did not find a significant relation between the magnitude of the decreases. Because the fMRI and MEG measures cannot be obtained in a simultaneous fashion, this finding partly reflects the presence of inter-subject and inter-session variability in resting-state brain connectivity. Differences in the experimental settings may have also been a factor (e.g., subjects were supine for the fMRI experiments but sat in a reclining chair for the MEG experiments). Prior work has demonstrated that there can be considerable variability across subjects and scans in the amplitude of the resting-state fMRI global signal, which is proportional to the average global connectivity (He and Liu, 2012; Wong et al., 2012). Variability in the degree of connectivity in the pre-dose state can alter the observed changes in connectivity. For example, if a subject has a typical level of resting-state connectivity (as compared to the rest of the sample) in the pre-dose MEG scan on one day but a lower relative level of connectivity in the pre-dose fMRI scan on another day, these differences in pre-dose connectivity will tend to lead to a relatively smaller reduction in this subject’s fMRI connectivity, as compared to the decrease in their MEG connectivity. Indeed, in the current study, we find that the relation between fMRI and MEG connectivity measures is considerably weakened by the relatively low pre-dose fMRI connectivity levels in two of the subjects. When considering the measures from the remaining eight subjects, we find a stronger (and nearly significant) relation between the fMRI and wide-band MEG connectivity measures.

As prior work has shown that fMRI fluctuations reflect a complex interaction of neuronal processing across different frequency bands (Mantini et al., 2007), we also examined the contribution of different bands to the observed wide-band MEG connectivity changes. We found that connectivity changes in the β band (both low and high) exhibited a significant effect of the caffeine/control factor and that changes in these bands were significantly correlated with the connectivity changes observed in the wide-band MEG signal. This finding is consistent with the growing body of resting-state literature which has shown a close relationship between β band oscillations and the BOLD signal in the motor cortex, visual cortex, and other resting-state networks (Brookes et al., 2011b; Stevenson et al., 2011). Furthermore Liu et al. (2010) found the large-scale synchrony of MEG power fluctuations (assessed at the sensor level) to be strongest in the β band and proposed that this finding suggested a neural basis for the global signal observed in resting-state fMRI.

Although considerably more informative then sensor measurements, the MEG beamformer approach used in this study has some potential limitations. Due to overlapping lead fields, signal leakage can occur and MEG time-courses from separate locations may appear to be correlated even though no true underlying FC exists (Brookes et al., 2011a). In this study, signal leakage effects were reduced by forming average time-courses from dipoles located within a sphere of 12 mm radius at the center of each ROI, thus minimizing the inclusion of physically adjacent dipoles from different ROIs. Prior work has shown that leakage is influenced by the choice of regularization as well as preprocessing artifact reduction steps (Brookes et al., 2008a,b). We verified that the degree of regularization performed in this study was consistent with that used in prior resting-state MEG connectivity studies (Brookes et al., 2011a,b; Luckhoo et al., 2012). Future connectivity studies could perhaps better address the leakage issue by applying techniques insensitive to leakage (Brookes et al., 2012b). Furthermore, an inherent limitation of the beamformer approach is its inability to resolve correlated sources, resulting in source suppression and time-course distortion (Sekihara et al., 2002). However, these limitations may be less pronounced in this study as the beamforming is performed on the filtered MEG time series while the correlation is computed using the MEG power fluctuations (Brookes et al., 2011a,b). Nevertheless, future implementation of techniques which have addressed the issue of correlated source suppression could be beneficial (Wipf et al., 2010; Diwakar et al., 2011).

In conclusion, this study demonstrates the similarity in caffeine-induced changes as assessed with both fMRI and MEG, supporting the neural origins of the BOLD connectivity decreases. This finding serves to provide a firmer basis for the use of fMRI as a tool for the evaluation of FC at the neural level. In addition, our results further demonstrate the utility of source-localized MEG measures for the assessment of resting-state connectivity.

## Conflict of Interest Statement

The authors declare that the research was conducted in the absence of any commercial or financial relationships that could be construed as a potential conflict of interest.
